# Endotracheal Tube Migration in Steep Trendelenburg Position With the Estape TrenMAX Positioning System

**DOI:** 10.7759/cureus.20664

**Published:** 2021-12-24

**Authors:** Marisol Alvarez, Sheila Llanes Rico, Jeffrey Tsai, Robin M Schaffer, Mohammed Masri, John Sciarra, Andrzej Kuchciak

**Affiliations:** 1 Anesthesiology, Larkin Community Hospital-South Miami, South Miami, USA; 2 Internal Medicine, Larkin Community Hospital-Palm Springs, Hialeah, USA; 3 General Surgery, Larkin Community Hospital-South Miami, South Miami, USA

**Keywords:** positioning in the or, academic anesthesiology, extreme trendelenburg position, trendelenburg, endotracheal tube, robotic-assisted laparoscopic gynecologic surgery, quality improvement and patient safety, obstetrics anesthesiologists, trenmax, perioperative complications

## Abstract

The extent of endotracheal tube (ETT) migration was studied in 25 adult female patients undergoing robotic-assisted laparoscopic gynecologic surgery while in a steep Trendelenburg position secured with the Estape TrenMAX positioning system (Innovative Medical Products, Plainville, CT, USA). In four patients, the distance from the tip of the ETT to the carina did not change. In three patients, the distance from the tip of the ETT to the carina decreased by 1 centimeter (cm). In other patients, the distance from the tip of the ETT to the carina decreased by 0.2 to 0.5 cm. We concluded that the tip of the ETT moves closer to the carina in patients put in the extreme Trendelenburg position. These results were in alignment with the evidence base created by other researchers.

## Introduction

Artificial airway placement and maintenance are critical activities performed daily by anesthesiologists in the operating room (OR). An endotracheal tube (ETT) is one of the most common airways for patients undergoing general anesthesia. The success of ETT placement depends on many factors such as patient age, sex, length of trachea, and the presence of edema, stenosis, or injury. Failure to advance the ETT to an appropriate extent will have significant clinical implications. An inadequately advanced ETT is as dangerous as one that is advanced too deep into the trachea [1]. The former increases the likelihood of accidental extubation during surgery; the latter can lead to endobronchial intubation, resulting in adverse events such as barotrauma [1,2]. For these reasons, anesthesiologists look for ways to reduce the extent of ETT migration or at least minimize the adverse effects of such migration. They may monitor ETT placement and migration through a variety of methods, including end-tidal carbon dioxide monitoring, thoracic ultrasound, and chest X-ray [3-5]. Various tapes and tube holders have been evaluated for their effectiveness in preventing ETT migration [6].

In addition to placing the ETT appropriately, anesthesiologists must also be aware of the physical forces and shifts that occur during surgery. The way the patient is positioned can greatly impact the process of ventilation [7]. One position of interest is the extreme Trendelenburg position, in which the patient lays supine at a 35-45 degree angle with their feet elevated above their head [8]. The position is mostly used for urologic and gynecologic surgery, such as robotic-assisted prostatectomy [7,8]. The position itself is known to cause numerous complications, from ocular injury to soft tissue edema [8].

One of the most significant risks of the extreme Trendelenburg position is that of unintentional movement, which may be so significant as to cause the patient to slip off the OR table [8]. Pisano provides a detailed description of the physics behind the Trendelenburg position. To keep the patient on the table, three forces must be in place - the force of the patient’s weight (or gravity), the force the patient exerts against the OR table, and the frictional force [8]. These forces counterbalance one another, keeping the patient on the table [8]. However, even when the risks of slipping off the table are minimal, the frictional force from both pneumoperitoneum and gravity may cause the ETT to migrate. 

The Trendelenburg position poses a variety of other challenges for the anesthesiologist. Because of the complex physical forces involved, this position incurs significant respiratory stress on patients, particularly those who are morbidly obese. Up to 22% of patients placed in the Trendelenburg position during surgery will experience limited expiratory flow and airway closure [9]. The Trendelenburg position also impairs normal respiratory mechanics, sometimes leading to pulmonary complications such as respiratory acidosis and atelectasis [10]. Both pneumoperitoneum and the Trendelenburg position cause a significant decrease in heart rate, which returns to normal as the operation proceeds but still requires close monitoring of both heart rate and cerebral oxygenation [11]. ETT migration may exacerbate these risks.

Current knowledge about ETT migration in the Trendelenburg position is scarce. The earliest published data on ETT migration in relation to patient positioning is from 1969. The Trendelenburg position was found to reduce the distance between the ETT tip and the carina by 0.3 to 0.6 centimeters (cm) when compared to the supine position [1]. Another study confirmed a mean reduction of 0.45 cm, with an even greater reduction after pneumoperitoneum [12]. When the patient undergoes both pneumoperitoneum and the Trendelenburg position, the mean reduction was found to be 0.85 to 1.71 cm [12,13]. Approximately half of the reduction is thought to be due to tracheal shortening [13].

Despite the growing body of literature, a more evidence-based understanding of ETT migration is needed when the patient is placed in the extreme Trendelenburg position. This could inform the provision of safer and more effective anesthesia while minimizing the risks of adverse patient events in a perioperative setting. This topic is even more important considering the rapid development of novel and highly technological solutions used for safe and effective patient positioning in the OR. The Estape TrenMAX (Innovative Medical Products, Plainville, CT, USA) is one of the latest and most innovative secure positioning systems available in the OR. The use of such positioning systems may affect the extent of ETT migration. This report aims to describe ETT movement in patients placed in the extreme Trendelenburg position while using Estape TrenMAX during robotic-assisted laparoscopic gynecologic surgery.

## Materials and methods

Project aim

**Figure 1:**
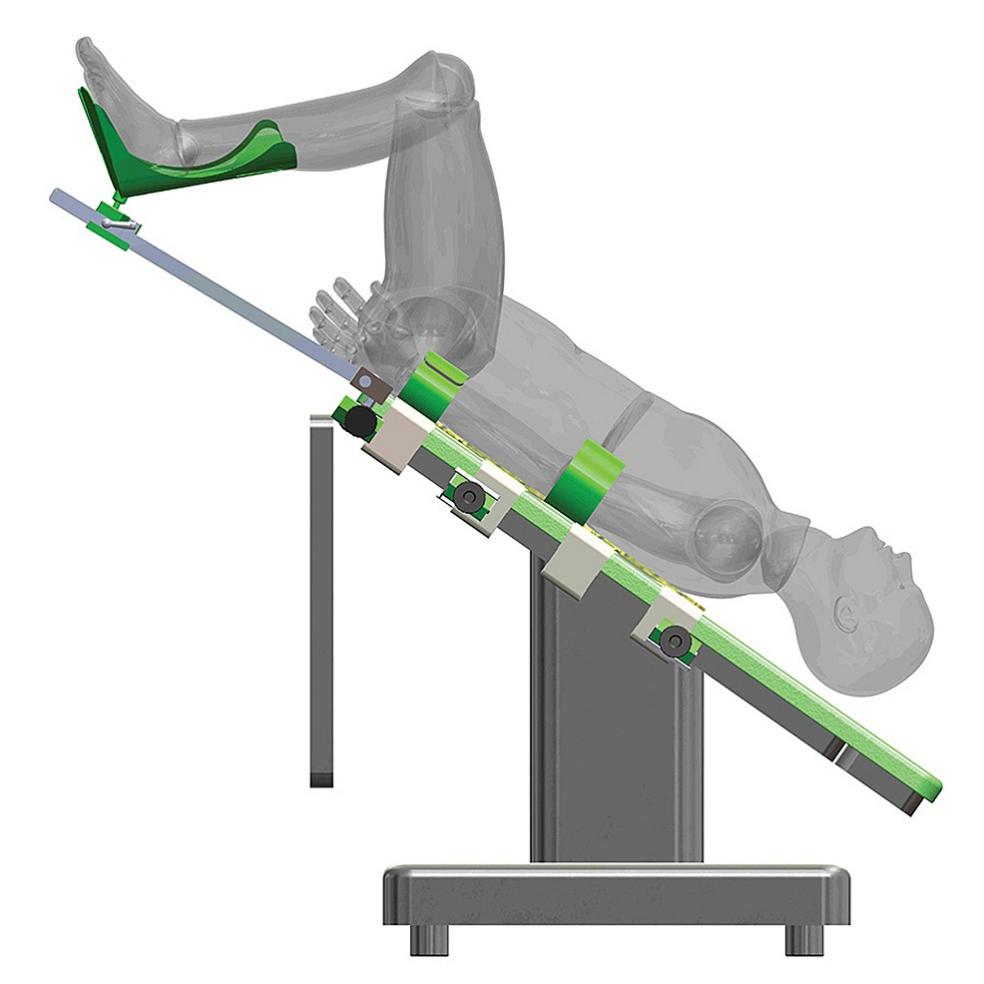
Estape TrenMAX system in extreme Trendelenburg position.

Estape TrenMAX (Figure 1) is a novel positioning system that allows for Trendelenburg and Reverse Trendelenburg positions without using conventional foam-based positioning pads [14]. With any new positioning system, anesthesiologists and surgeons should be cautious about the perioperative impact on ETT migration to ensure patient safety. This project aimed to pioneer efforts to assess ETT migration during extreme Trendelenburg position when using Estape TrenMAX in robotic-assisted laparoscopic gynecologic surgery, such as myomectomy and hysterectomy [15].

Setting

This clinical project was carried out at Larkin Community Hospital (LCH) in South Miami, Florida. LCH is a 146-bed for-profit medical facility providing a broad spectrum of services, including gynecologic oncologic surgery. With approximately 1,500 inpatient and outpatient surgeries annually, the hospital offered ample opportunity to complete this project. Innovative Medical Products Inc. (IMP) markets the Estape TrenMAX with a proprietary Sticky Pad that adheres to the patient’s body. The Sticky Pad has "hook-and-loop" tightening components that are strapped to the side rails of the OR table and secured with clamps in order to prevent any unnecessary patient movement during robotic-assisted laparoscopic gynecologic surgery [15]. 

Criteria of inclusion and exclusion

Female patients between the ages of 21 and 79 years old were included in this study if they were scheduled to undergo robotic-assisted laparoscopic gynecologic surgery at LCH for two years. The IRB Analyst/Coordinator for Department of Research & Academic Affairs issued approval LCH-8-072019. All patients received information about this project in layman’s terms, both in writing and verbally, on the day of admission. All patients were assured that no confidential, personal or identifiable information would be needed. No rewards or incentives were offered to patients for their participation. All respondents provided written informed consent before the assessments. Patients were excluded from the analysis if they were unable to tolerate the extreme Trendelenburg position in any physical or physiological challenge.

Stepwise assessments

**Figure 2:**
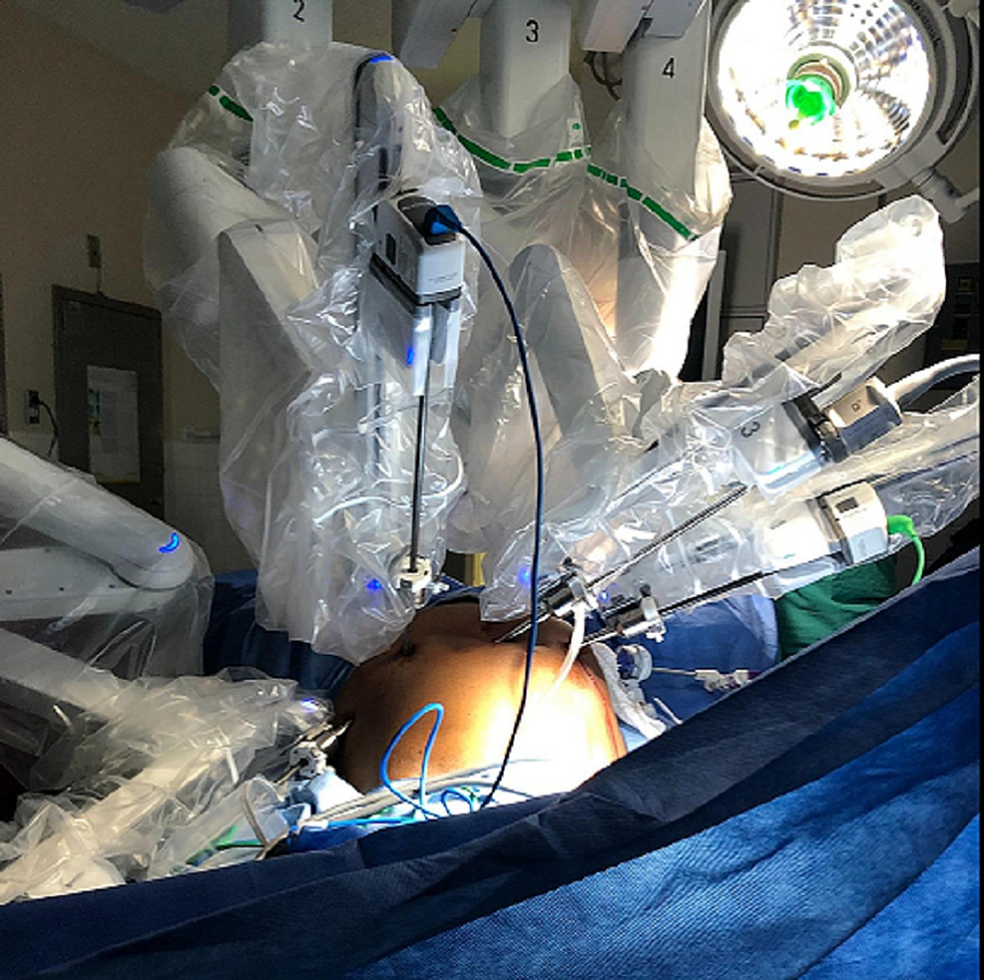
Estape TrenMAX system during robotic-assisted laparoscopic gynecologic surgery

1. An OR table was prepared with Estape TrenMAX basic padding in advance. 2. The patient was transported to the OR, where they were properly sedated and intubated by the anesthesia team. 3. The OR nurses securely positioned the patient on the Estape TrenMAX by following the instructions from the manufacturer [15]. 4. With the patient still in the supine position, the anesthesia team inserted a flexible fiberoptic bronchoscope into the tracheal lumen of the ETT to measure the distance between the distal tip of the ETT and the carina. 5. After the flexible fiberoptic bronchoscope was removed, the patient was securely positioned in the extreme Trendelenburg position. 6. With the patient in Trendelenburg position, the same anesthesia team then inserted the same flexible fiberoptic bronchoscope once again into the patient’s tracheal lumen of the ETT to measure the distance between the distal tip of the ETT and the carina. 7. After the flexible fiberoptic bronchoscope was removed, the patient was returned to a supine position to allow the surgeon to place robotic laparoscopic ports and to insufflate the abdomen. 8. After the surgeon’s confirmation of surgical placements, the patient was again positioned in the extreme Trendelenburg position (Figure 2). 9. 15 minutes later, the same anesthesia team inserted the same flexible fiberoptic bronchoscope once again into the patient’s tracheal lumen of the ETT to measure the distance between the distal tip of the ETT and the carina. 10. At the end of the surgery, the patient was properly extubated without complications by the same anesthesia team. 11. All steps above were implemented with equipment and time-controlled to avoid any unnecessary delay. The patients were monitored for hemodynamic stability.

Data collection

The following data were collected: patient gender, age, height, weight, ETT measurement at the level of teeth (pre- and post-Trendelenburg), ETT distance to the carina (pre- and post-Trendelenburg), ETT size, and shoulder position was marked (pre- and post-Trendelenburg).

Data analysis

Descriptive statistics were used to measure the minimum and maximum ETT migration distance in patients put in the extreme Trendelenburg position and the mean change in the ETT-carina distance.

## Results

Findings

The sample included 25 patients admitted for robotic-assisted laparoscopic gynecologic surgery at LCH. The mean age was 50 years (SD 11.8) (Table 1). The youngest patient was 21 years old, and the oldest was 79 years old. All patients were female (N=25; 100%). All patients were admitted for robotic-assisted laparoscopic gynecologic surgery (N=25; 100%). No other demographic characteristics were collected.

**Table 1:** Patients' Demographic Characteristics

Demographic characteristics		N=25
Age (years)		
	Mean	50.32 years
	SD	11,824
	MIN	21
	MAX	79
Gender		
	Male	0
	Female	25 (100%)
Type of surgery		
	Robotic-assisted laparoscopic gynecologic surgery	25 (100%)

ETT size ranged between 7.0 and 7.5 in all patients. ETT measurement at the teeth ranged between 20 and 23 cm (Table 2). ETT at the level of teeth did not change during the surgery; the ETT-teeth distance was the same pre-and post-surgery in each of the 25 patients included in the sample.

**Table 2:** Distance in cm of ETT-teeth and ETT-carina distance between pre-Trendelenburg and post-Trendelenburg positioning ETT: endotracheal tube

Data		Pre-Trend	Post-Trend	Change/migration
ETT-teeth distance (cm)				
	Min	20	20	0
	Max	23	23	0
	Mean	21.76 (SD=0.72)	21.76 (SD=0.72)	None
ETT-carina distance (cm)				
	Min	1.5	0.5	0
	Max	3.5	3	1
	Mean	2.6 (SD=0.59)	2.3 (SD=0.63)	0.376 (SD=0.29)

**Figure 3:**
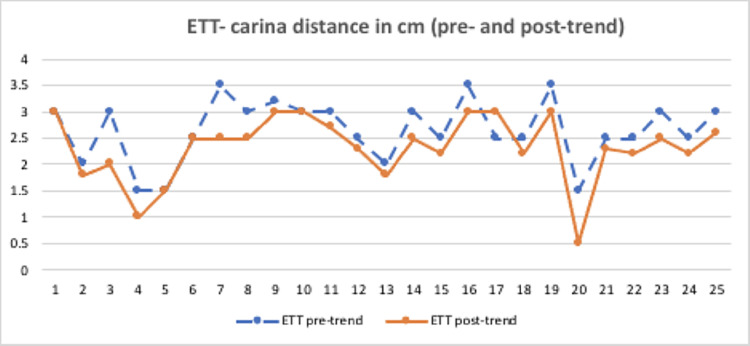
Statistical diagram of the changes in ETT-carina distance between pre-Trendelenburg and post-Trendelenburg positioning X axis = Each of the 25 patients in this project; Y axis = ETT-carina distance ETT: endotracheal tube

The distance between the ETT and the carina (or “ETT-carina distance”) differed significantly from pre-Trendelenburg to post-Trendelenburg (Figure 3). The mean change in the ETT-carina distance was 0.376 cm (Table 2). In four patients, the ETT-carina distance did not change (0 cm). In three patients, the ETT-carina distance decreased by 1 cm. In other patients, the ETT-carina distance decreased by 0.2 to 0.5 cm. The shortest ETT-carina distance was 1.5 cm pre-Trendelenburg and 0.5 cm post-Trendelenburg. The longest distance was 3.5 cm pre-Trendelenburg and 3 cm post-Trendelenburg.

## Discussion

Today, anesthesiologists and surgeons have access to advanced technologies that maintain patients in a relatively safe extreme Trendelenburg position while keeping unnecessary movements to a minimum. To the researchers’ knowledge, this is the first research project of this kind that describes ETT migration when using the Estape TrenMAX for patients placed in the extreme Trendelenburg position during robotic-assisted laparoscopic gynecological surgery.

In this study, the mean shortening of the ETT-carina distance was 0.376 cm. This amount of ETT migration was within the range found in previous studies of patients in the Trendelenburg position, in whom the mean shortening was found to be between 0.3 and 0.6 cm [1,12]. Meanwhile, it was less migration than found in one study in which the extent of ETT migration was between 0.4 and 1.8 cm [5]. ETT migration is often significant after abdominal insufflation, ranging from 0.7 to 1.71 cm [1,12,13]. It is difficult in retrospect to identify and evaluate all the factors responsible for ETT migration in former studies. Although the data obtained in this research project is comparable to the data reported in other sources, exact comparisons are difficult due to the scarcity of evidence and statistical data on ETT migration in patients placed in the extreme Trendelenburg position [1,5].

The common finding among these studies is that the tip of the ETT moves closer to the carina in patients put in the extreme Trendelenburg position. Although the current research into ETT migration in patients undergoing surgery in this position is scarce, these results fit in the evidence-based picture created by other researchers [1,5,8]. The three forces affecting patients in the Trendelenburg position also cause frictions that explain ETT migration during surgery [8]. Still, more research is needed to evaluate the effect of each of these physical forces on ETT migration and explain the mechanism behind changes in the ETT-carina distance in patients undergoing gynecologic surgery in the extreme Trendelenburg position.

This study has several limitations. First, it was conducted at a single institution. Second, the sample was small and limited to women undergoing robotic-assisted laparoscopic gynecologic surgery. Because of this, the results might not be generalized to other populations, such as men undergoing robotic-assisted laparoscopic prostatectomy. Third, several factors that could potentially moderate or confound the effect of the extreme Trendelenburg position on ETT migration were not considered, such as patient age, body mass index, and ETT size. Finally, the study did not include a control group for comparison.

Future research is needed to address two major knowledge gaps. The first is to explore all the factors influencing the extent of ETT migration in patients undergoing surgery in the extreme Trendelenburg position. The second is to compare the differences in ETT migration in patients placed in different positions. Both will enrich anesthesia practices, particularly in light of the technological advances and novel devices used to facilitate surgery and improve patient outcomes.

## Conclusions

ETT migration is a challenge for anesthesiologists managing patients in the Trendelenburg position. With the results of this study, anesthesiologists will be better prepared to monitor and manage ETT migration in patients undergoing robotic-assisted laparoscopic gynecologic surgery and perhaps other surgeries requiring the Trendelenburg position. A better understanding of ETT migration during surgery may reduce the risks of adverse outcomes such as endobronchial intubation. Future research is needed to quantify the effects of certain factors on ETT migration, such as patient weight and BMI, ETT size, and the angle at which the patient is tilted while on the operating table. Future studies should also compare the extent of ETT migration between patients using the Estape TrenMAX system and those placed on standard foam pads.
